# Ultrasonic Nanobubbles Carrying Anti-PSMA Nanobody: Construction and Application in Prostate Cancer-Targeted Imaging

**DOI:** 10.1371/journal.pone.0127419

**Published:** 2015-06-25

**Authors:** Xiaozhou Fan, Luofu Wang, Yanli Guo, Zhui Tu, Lang Li, Haipeng Tong, Yang Xu, Rui Li, Kejing Fang

**Affiliations:** 1 Department of Ultrasound, Southwest Hospital, Third Military Medical University, Chongqing, China; 2 Department of Urology, Daping Hospital, Institute of Surgery Research, Third Military Medical University, Chongqing, China; 3 State Key Laboratory of Food Science and Technology, Nanchang University, Nanchang City, Jiangxi Province, China; 4 Jiangxi-QAI Joint Research Institute, Nanchang University, Nanchang City, Jiangxi Province, China; Vrije Universiteit Brussel, BELGIUM

## Abstract

To facilitate prostate cancer imaging using targeted molecules, we constructed ultrasonic nanobubbles coupled with specific anti-PSMA (prostate specific membrane antigen) nanobodies, and evaluated their *in vitro* binding capacity and *in vivo* imaging efficacy. The “targeted” nanobubbles, which were constructed via a biotin-streptavidin system, had an average diameter of 487.60 ± 33.55 nm and carried the anti-PSMA nanobody as demonstrated by immunofluorescence. Microscopy revealed targeted binding of nanobubbles *in vitro* to PSMA-positive cells. Additionally, ultrasonography indicators of nanobubble imaging (including arrival time, peak time, peak intensity and enhanced duration) were evaluated for the ultrasound imaging in three kinds of animal xenografts (LNCaP, C4-2 and MKN45), and showed that these four indicators of targeted nanobubbles exhibited significant differences from blank nanobubbles. Therefore, this study not only presents a novel approach to target prostate cancer ultrasonography, but also provides the basis and methods for constructing small-sized and high-efficient targeted ultrasound nanobubbles.

## Introduction

The specific identification of prostate cancer is a clinically urgent task [1]. In this regard, the development of ultrasound molecular imaging has provided a new avenue for early prostate cancer diagnosis. This technique involves labeling imaging compounds with specific antibodies or ligands to generate targeted ultrasound contrast agents capable of binding to specific tissues or lesions. After intravenous administration, these molecular probes aggregate specifically in the target tissues via the blood circulation, thus allowing ultrasonography-based specific imaging of pathogenic changes at a molecular or cellular level. [2]. However, the micron-scale ultrasound contrast agents (microbubbles) currently used in most relevant imaging studies have diameters of 1–10 μm [3,4]. Tumor neovascular structures are often imperfect because tumor blood vessels feature incomplete basement membranes, lack smooth muscle layers and exhibit poor lymphatic circulation; accordingly, these vessels exhibit increased permeability relative to normal blood vessels, an effect that has been termed the enhanced permeability and retention effect (EPR). Despite this permeability, the maximal vascular pore diameter ranges from approximately 380–780 nm, and theoretically only particles <700 nm in diameter can pass through the tumor neovascularization; therefore, regular ultrasound contrast agents often cannot pass through the vasculature to research tumor cells and facilitate specific tumor imaging [5,6]. Following these EPR findings, some groups have recently constructed nanobubbles and examined their permeability. The nanobubbles prepared by Yin *et al*. had an average diameter of 436.8 ± 5.7 nm and displayed passive tumor targeting [7]. In our preliminary studies, we also developed targeted nanobubbles with an average diameter of 644.30±55.85 nm that carried monoclonal antibodies against prostate-specific membrane antigen (PSMA) and investigated the imaging potential of these nanobubbles in prostate cancer xenografts in nude mice. Our results revealed that these targeted nanobubbles could augment the xenograft peak time and intensity values compared with blank nanobubbles and were therefore conducive to tumor-specific targeted imaging [8].

Nevertheless, monoclonal antibody-targeted nanobubbles have two apparent limitations. First, murine monoclonal antibodies are immunogenic in humans. Second, the large molecular weights of antibody-particle complexes result in low numbers of targeting complexes reaching the intended targets [9], thereby compromising the imaging outcomes. Hence, the identification of a small-sized antibody that is convenient, high-efficient and penetrating is crucial for tumor-targeted molecular imaging. The discovery of nanobodies [10] provided a promising strategy to develop a new type of ultrasound-targeted nanobubble because these nanobodies are smaller in size. Specifically, IgG2 and IgG3 (heavy chain antibodies) from animals in the Camelidae family naturally lack light chains and the CH1 domain; these are the smallest functional currently known antigenic binding fragments and are characterized by low molecular weights, convenient expression, stability and low immunogenicity *in vivo*. Hence, nanobodies are a promising prospect with respect to accurate diagnosis and targeted therapies [11–16]. However, very few tumor-targeted ultrasound nanobubbles coupled with specific nanobodies were described. In the present work, we developed the small-sized and high-efficient targeted nanobubble formulation, which carried the anti-PSMA nanobody, to verify the hypothesis that nanobody-coated nanobubbles can enhance the diagnostic value of ultrasound in prostate cancer.

## Materials and Methods

### Cells and animals

LNCaP, which is the typical human androgen-dependent prostate cancer cell, was purchased from the American Type Culture Collection (ATCC). C4-2, which is a subtype of the LNCaP cell line and a human androgen-independent prostate cancer cell line, was acquired from ViroMed Laboratories at Johns Hopkins, USA. MKN45, a human gastric cancer cell line as the control, was obtained from the Chinese Academy of Medical Sciences Cancer Institute (Beijing, China). Four- to 5-week-old male BALB/c-nu nude mice (Experimental Animal Center, Third Military Medical University) kept in a specific pathogen-free environment were used as described below. All animal experiments were approved by the Animal Ethics Committee of The Third Military Medical University.

### Western blotting in three cell lines

LNCaP, C4-2 and MKN45 cells were grown to the logarithmic phase, rinsed with phosphate-buffered saline (PBS), placed on ice, and suspended in 400 μl of radioimmunoprecipitation assay (RIPA) protein lysis buffer. Next, all tumor cell lysates were transferred to a 1.5-mL tube and centrifuged at 15000 rpm and 4°C for 15 min. The resulting supernatant was transferred to a new 1.5 mL centrifuge tube. A bicinchoninic acid (BCA) kit was then used to determine the protein concentration. Additionally, the samples were supplemented with 5X sodium dodecyl sulfate-polyacrylamide gel electrophoresis (SDS-PAGE) loading buffer, mixed and boiled for 5 min to fully denature the proteins. Thirty micrograms of total protein was separated via SDS-PAGE and transferred to a polyvinylidene fluoride (PVDF) membrane via the semi-dry blotting method. The membrane was blocked with a 5% skim milk buffer at room temperature for 2 h and was then probed successively with a 1:400 dilution (2.5 μg/mL) of an anti-hPSMA monoclonal antibody at 4°C overnight and a 1:2000 dilution of a horseradish peroxidase (HRP)-conjugated secondary antibody at room temperature for 2 h; the membrane was washed three times with PBST (PBS with 0.1% Tween-20) after each antibody incubation.

### Generation of specific and biotinylated nanobody

The extracellular region of PSMA was first expressed in eukaryotic human embryonic kidney (HEK)-293 cells, after which the recombinant protein was used as the coating material to screen a previously established natural nanobody library designated NA-PDL. Correspondingly, nanobody phages capable of specifically binding to PSMA at the molecular and cellular levels were obtained [17]. These phages were subsequently sequenced, and the following primers were designed according to the sequencing results: forward primer, CGCGGATCCATGGCCCAGGTGCAGCTGGTG (containing a *BamHI* site) and reverse primer, CCCAAGCTTTTATTGTGGTTTTGGTGTCTTGGGTT (containing a *HindⅢ* site). A polymerase chain reaction (PCR) was then performed, using the positive phage clone as a template to amplify the target gene; the reaction product was subsequently cloned into the *BamHI* and *HindIII* sites of the pET28a expression vector (Novagen/EMD Millipore, Billerica, MA, USA), which contains a six-histidine tag. The recombinant vector was transformed into the *E*. *coli* DH5α strain. The resulting positive clones were sequenced to identify those with the correct sequence; the correct clones were transformed into the *E*. *coli* Rosseta expression strain (DE3; Novagen/EMD Millipore) to yield a high expression level. Ni-Agarose (Qiagen, Venlo, The Netherlands) was subsequently used to purify the histidine-tagged nanobody. Next, we labeled the nanobody with the solution of biotin. In detail, two milligrams of Sulfo-NHS-LC-Biotin (Pierce/Thermo Scientific, Rockford, IL, USA) were fully solubilized in 360 μL of sterile ddH_2_O. This solution was incubated with the nanobody at 4°C for 72 h, followed by dialysis at 4°C overnight. UV spectroscopy was used to determine the antibody concentration. Specifically, the theoretical extinction coefficient from the sequence of the nanobody was 21555 M^-1^·cm^-1^, and the absorbance at 280 nm was measured to calculate the antibody concentration according to the formula “Absorbance = ε (extinction coefficient, M^-1^·cm^-1^) X pathlength (cm) X concentration (M)”. A biotin quantification kit (Pierce/Thermo Scientific) was used to calculate the biotin concentrations in the samples and generate the biotin/antibody conjugation ratio

### Validation of the nanobody affinity via enzyme-linked immunosorbent assay (ELISA)

To obtain the affinity of the biotinylated nanobody, a standard competitive ELISA was used. Every well of a microtitre plate was coated with 1 mM recombinant PSMA antigen, blocked with 3% bovine serum albumin (BSA)-PBST at room temperature for 2 h and then rinsed three times with PBST. Next, 1 nM biotinylated nanobody was incubated with increasing concentrations of antigen at concentrations ranging from 0.1 nM to 100 μM in parallel eppendorf tubes. After 30 minutes incubation, 90 μL of the reaction mixtures were applied to the wells of the antigen-coated microtitre plate. After 10 min incubation, the mixtures were discarded, and the wells were rinsed with PBST. Next, 100 μL of HRP-streptavidin conjugated-biotin (Kangwei Century, Beijing, China) at a 1:2000 dilution was added to each well, followed by incubation at 37°C for 1 h. Every well was then rinsed 5 times with PBST before adding 100 μL/well of a 3,3’,5,5’-tetramethylbenzidine (TMB) working solution (Beyotime, Shanghai, China) and incubating the plate at room temperature for 15 min. The reactions were terminated by adding 50 μL of a 2 M sulfuric acid solution to each well. The absorbance at 450 nm was subsequently determined for each well. Therefore, the highest optical density (OD)_450nm_ should have been observed at low concentrations of antigen. The concentration of antigen at which the half-maximal ELISA signal is detected corresponds to the dissociation constant K_D_.

### Preparation and validation of targeted nanobubbles

Mixtures containing specific ratios of dipalmitoyl phosphatidyl choline (DPPC; Genzyme Pharmaceuticals, Bromma, Sweden), biotinylated distearoyl phosphatidyl ethanolamine (Bio-DSPE; Avanti Polar Lipids, Inc., Alabaster, AL, USA) and diphenylphosphoryl azide (DPPA; Lipoid GmbH, Ludwigshafen, Germany) were weighed and lyophilized using a freeze dryer (Shanghai Pudong Freeze Drying Equipment Co., Shanghai, China). Aliquots of these mixtures were placed into vials; octafluoropropane (C_3_F_8_) gas was slowly injected to replace the air overlays in the vials. Prior to use, a mixed solution of 1 part glycerol: 9 parts PBS was added to the vial followed by warming to 37°C to facilitate solubilization. The preparations were horizontally mixed in a reverse manner using a ST series amalgamator (AT&M Biomaterials Co., LTD, Beijing, China) with the following specific working parameters: vibration frequency, ≥4500/min; vibration amplitude, 15 ± 1 mm and vibration duration, 60 s [8]. These preparations were subsequently allowed to rest at 4°C to facilitate phase separation. The lower-phase milky suspension was centrifuged at 300 rpm for 3 min to separate the biotinylated nanobubbles at the bottom from the microbubbles at the top. Next, 3 μg of avidin were added per 10^7^ nanobubbles, followed by incubation at 4°C for 1 h. The preparations were allowed to rest to facilitate phase separation; the top layer was subsequently collected and centrifuged at 300 rpm for 3 min. The sample was rinsed three times to remove excessive avidin. Next, 1 μl of biotinylated nanobody was added per 10^7^ nanobubbles, followed by incubation, centrifugation and rinsing to remove the excess biotinylated nanobody. The resulting nanobubbles were designated the Targeted NBs, whereas nanobubbles without antibody supplementation were designated the Blank NBs. The particle sizes of the 2 products were analyzed on a Malvern Zetasizer nano ZS90 analyzer (Malvern Instruments Ltd., Malvern, UK), and a counting chamber was used to determine the concentrations of the 2 products. their *in vitro* imaging effects at the different concentrations were investigated with an agarose model under the condition of a mechanism index of 0.12 and a gain of 60%.

To investigate whether this method could be used to attach biotinylated nanobody to the nanobubbles, Blank or Targeted NBs were incubated with a mouse anti-His antibody for 1 h, followed by centrifugation, rinsing and incubation in the dark with a fluorescein isothiocyanate (FITC)-conjugated goat anti-mouse antibody for 3 h at 4°C. The samples were centrifuged and rinsed to remove unbound secondary antibody. The samples were then observed under a fluorescence microscope (Olympus Corporation, Tokyo, Japan) to evaluate the fluorescent binding.

### Nanobubbles and the *in vitro* cell binding assay

LNCaP, C4-2 and MKN45 cells grown to the logarithmic phase were centrifuged and seeded onto coverslips in the wells of a 24-well plate at a density of 1.5 × 10^4^ cells/well. The cells were cultured overnight, fixed with 4% paraformaldehyde and rinsed three times with PBS. Two groups of coverslips were prepared per cell type; 1 group was supplemented with 30 μl of Targeted NBs (1.0 x10^8^/mL) and the other with Blank NBs, after which the mixtures were incubated at 4°C for 3 h. Subsequently, the coverslips were rinsed three times with PBS, placed face-down onto slides and observed under a light microscope (Olympus Corporation) to examine the nanobubbles. The adhesion percentage, defined as the percentage of cells labeled with ≥4 targeted nanobubbles in a given random field, was calculated to indicate binding. This experiment was repeated 4 times.

### Nanobubble imaging in different xenografts

Logarithmic-phase LNCaP prostate cancer cells and C4-2 cells were used to prepare cell suspensions at a density of 5 × 10^7^ cells/mL; MKN45 gastric cancer cells in logarithmic phase growth were used to prepare cell suspensions of 1 × 10^7^ cells/mL. Two hundred microliters of each cell suspension were subsequently mixed with 200 μL of BD Matrigel (BD Biosciences, USA); the resulting mixtures were subcutaneously inoculated into 4–5-week-old male BALB/c-nu nude mice with a body weight of 18–20 g. Five animals were used for each xenograft tumor type. The xenografts were monitored daily with a vernier caliper until the tumors reached an approximate diameter of 1 cm.

Tumor-bearing nude mice were anesthetized via intraperitoneally administered 1% sodium pentobarbital. For imaging, the surfaces of both the probe and tumor were covered with a 5-mm thick coupling agent. A 50-mm L12-5 broadband linear ultrasound probe connected to an iU22 ultrasound system (Philips, Amsterdam, The Netherlands) was used to perform B-mode ultrasound imaging of the xenografts. Once the cross-section of a xenograft was fully revealed, the probe was immobilized to allow set-up of the ultrasonography mode (mechanical index, 0.12; gain, 90%). Ultrasonography was initiated when the focal center was positioned at the tumor center. Each test animal received 200 μL Blank or Targeted NBs (3 × 10^7^ particles) via tail vein injection, after which the pipeline was flushed with 100 μL of saline. Dynamic images were collected until the tumor area was completely free of NBs. Additionally, 200 μL of an equivalent amount of another type of nanobubbles and 100 μL of saline were administered in a similar manner to facilitate ultrasonography under identical conditions; the imaging data collected from the same nude mice were analyzed using Qlab8.1 software (Philips) to compare the 4 imaging parameters (arrival time, peak time, peak intensity and enhanced duration) in the xenograft area with the 2 contrast agents. The arrival time was defined as the interval from injection completion and the first time point at which 10% peak intensity was achieved. Enhanced duration was defined as the interval between the 2 time points at which 10% peak intensity was achieved. Peak time was defined as the interval between the injection time and the time of peak intensity [18].

### Statistical analyses

Statistical Package for Social Science (SPSS) 16.0 software (SPSS Inc., Chicago, IL, USA) was used to perform the statistical analyses. All of the quantitative data were expressed as means ± standard deviations. The Targeted NB and Blank NB ultrasound indicator data in the *in vitro* and *in vivo* imagings were obtained and analyzed using a paired sample T-test. The ultrasound indicators of the nanobubbles that targeted the 3 xenograft types were analyzed using an analysis of variance (ANOVA). A P-value <0.05 was considered statistically significant. Histograms and the curve with non-linear regression were plotted using GraphPad Prism 5.0 (GraphPad Software, La Jolla, CA, USA).

## Results

### PSMA expression in different cell lines and preparation of the PSMA-specific nanobody

**Fig 1:**
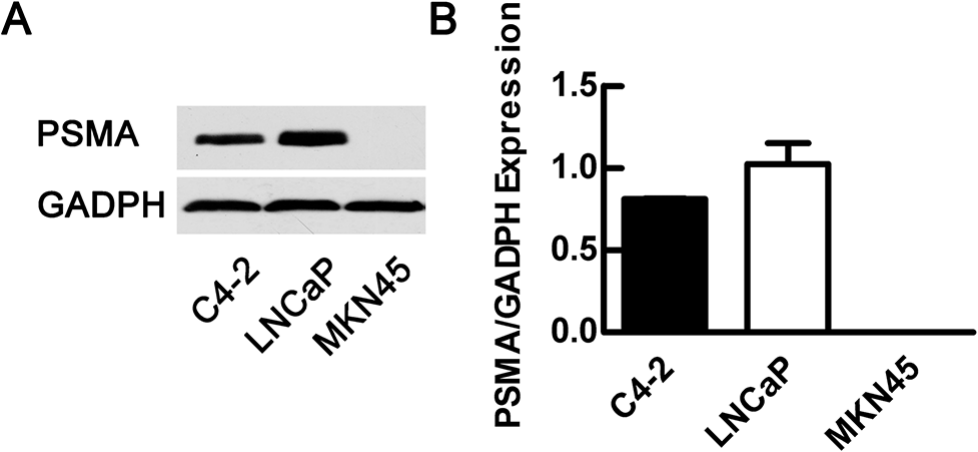
PSMA expression in the three cell lines (LNCaP, C4-2 and MKN45 cells) detected by Western blot. LNCaP cells expressed a higher level of PSMA than C4-2 cells, whereas MKN45 cells exhibited no expression.

Western blotting was used to examine PSMA expression in LNCaP, C4-2 and MKN45 cells. The results showed that among these cell lines, LNCaP cells expressed the highest level of PSMA, followed by C4-2 cells; MKN45 gastric cancer cells exhibited no apparent PSMA expression (Fig 1).

**Fig 2:**
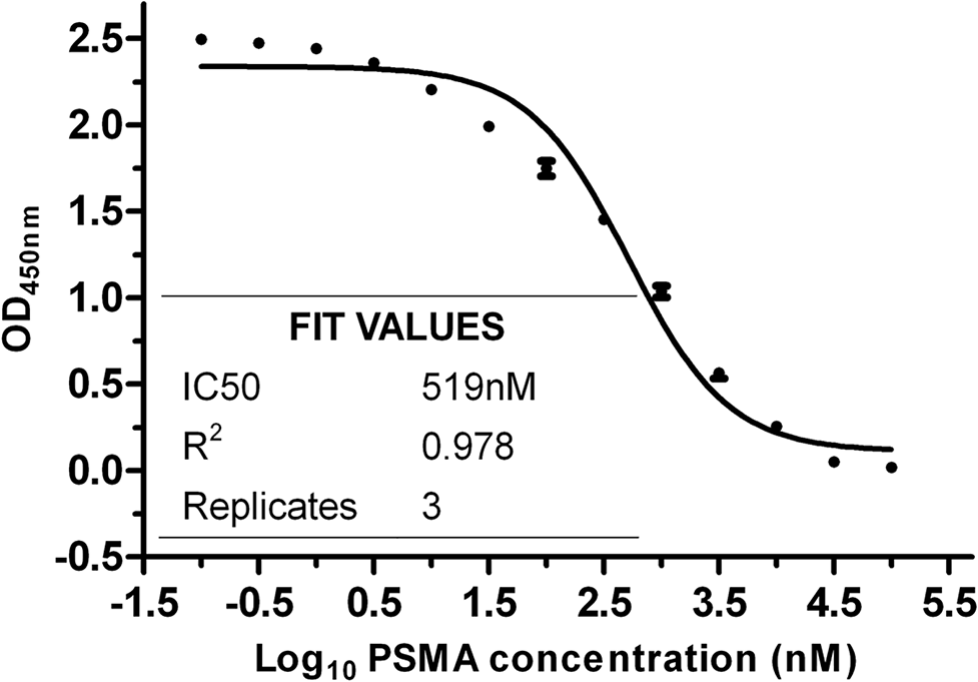
Evaluation of binding affinity of biotinylated nanobody with PSMA using the competitive ELISA. The curve with non-linear regression about the concentration of recombint PSMA ranging from 0.1 nM to 100 μM are obbtained.

The phage clone selected via prokaryotic panning was used for recombinant expression and facilitated the acquisition of His-tagged PSMA-specific nanobody (molecular weight, 18 kD). After biotinylating the nanobody, UV spectrometry was performed to reveal that the antibody concentration was 0.59 mg/ml and the biotin/nanobody conjugation ratio was about 3.6:1 according to a biotin quantification kit. Based on the principle of competitive binding, the dissociation constant is equal to the concentration at half-maximal value in ELISA. Therefore, the K_D_ of biotinylated nanobody against recombinant PSMA was 519 nM, while the non-linear regression had a good fitting with R^2^ = 0.978 (Fig 2).

### Targeted NB Characterization by fluorescence microscopy

**Fig 3:**
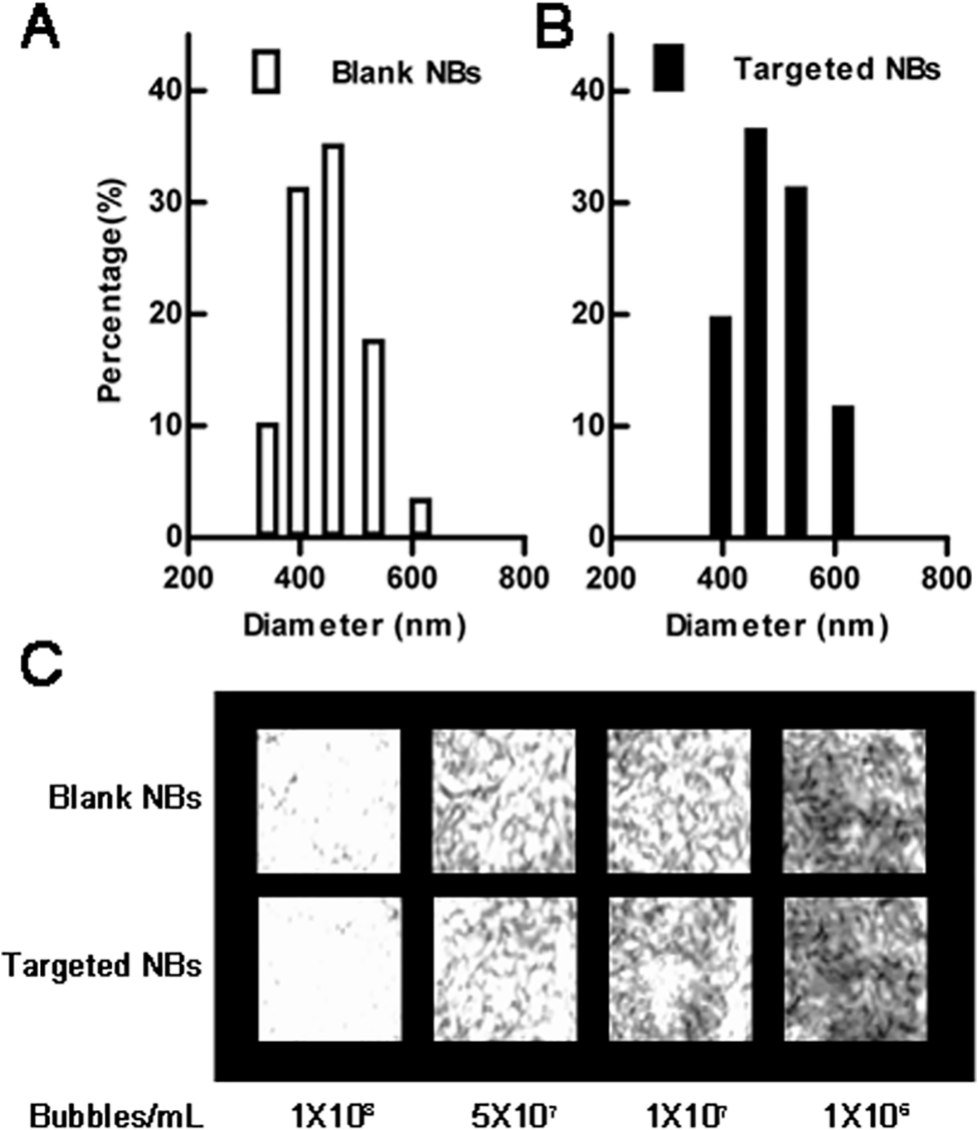
Particle size distributions and *in vitro* imaging of the Blank NBs and Targeted NBs. A Malvern Zetasizer analyzer was used to examine the particle sizes. Blank NBs (without biotinylated nanobody) had an average diameter of 445.30 ± 32.96 nm (A), whereas Targeted NBs (with biotinylated nanobody) had an average diameter of 487.6 ± 33.55 nm (B). And there is no difference in their *in vitro* imaging at the same concentrations (C).

**Fig 4:**
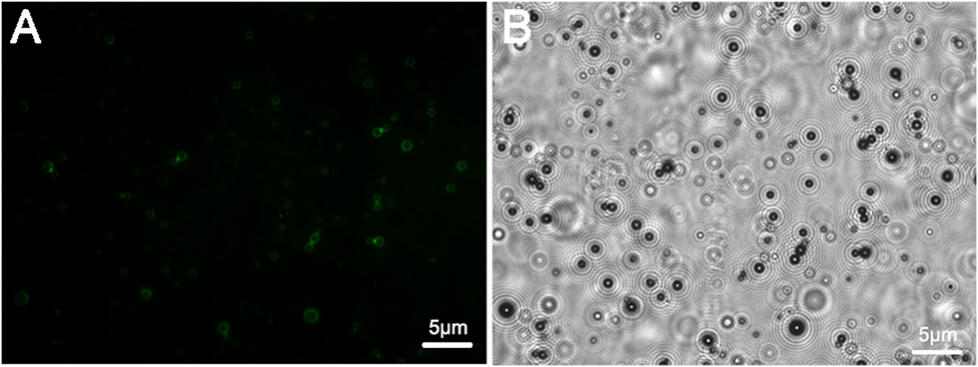
Immunofluorescence validation of the Targeted NBs. Microscopic observation of the Targeted NBs (A and B). The ring-like structures with thick membranes are NBs (B). The results demonstrated that Targeted NBs could specifically incorporate nanobody via biotin-avidin interactions.

The nanobubbles and biotinylated nanobody were conjugated with streptavidin-biotin. A light microscope and Malvern Zetasizer nano ZS90 analyzer were used to study the morphologies and diameters of the Targeted NBs and Blank NBs and revealed that both exhibited regular spherical shapes, and the Targeted NB preparation had an average diameter of 487.60 ± 33.55 nm, whereas the Blank NB preparation had an average diameter of 445.30 ± 32.96 nm; their *in vitro* imaging effects, namely ultrasound signals, had no differences at the same concentration (P = 0.06, Fig 3). Immunofluorescence later revealed that the targeted NBs emitted green fluorescence signals under the microscope (Fig 4). In contrast, the Blank nanobubbles, which were not labeled with biotinylated nanobody, did not display an apparent fluorescence signal, indicating that the nanobubbles specifically bound the nanobody via biotin-avidin interactions.

### Binding of targeted nanobubbles to cells

**Fig 5:**
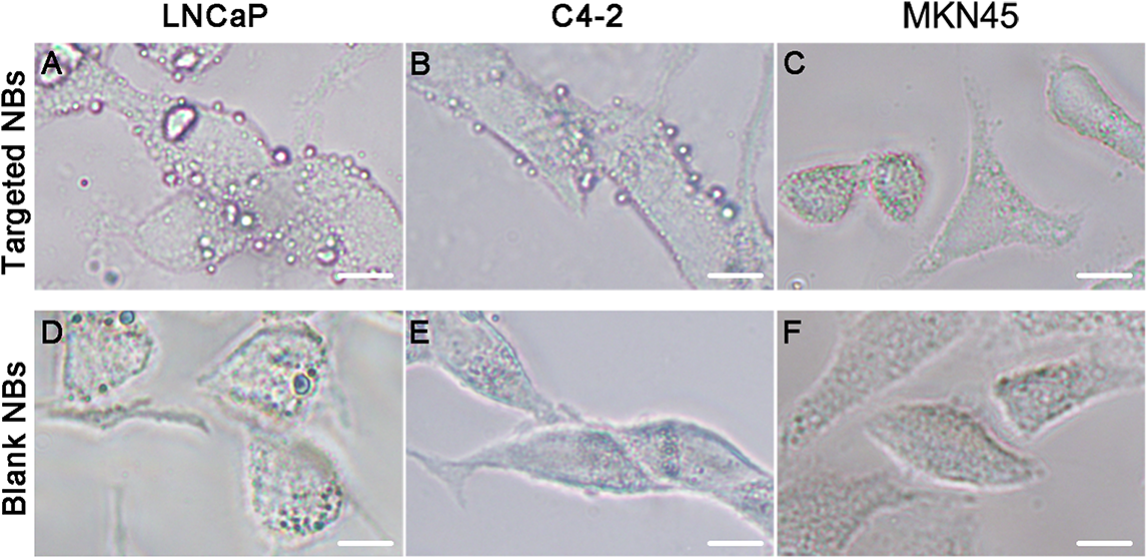
Binding of 3 types of cells to Blank NBs or Targeted NBs. Under a light microscope, both LNCaP cells and C4-2 cells visibly bound to Targeted NBs (A and B), whereas MKN45 cells did not bind to Targeted NBs (C). None of the 3 types of cells displayed prominent binding to Blank NBs (D, E and F). Scale, 5 μm.

Cell-nanobubble binding was examined via microscopy (Fig 5). The targeted nanobubbles adhered best to the LNCaP cells, each of which recruited an average of 7.27 ± 1.70 nanobubbles to yield an adhesion percentage of 98.00 ± 2.31%, followed by C4-2 cells, each of which recruited 5.67 ± 1.61 nanobubbles to yield an adhesion percentage of 92.00 ± 8.64%. In contrast, no significant binding was observed between the nanobubbles and MKN45 cells, with a binding frequency of 0.72 ± 0.87 nanobubbles per cell. Finally, no apparent binding was identified between the Blank NBs and each of the 3 types of cells, as evidenced by the corresponding binding numbers of 0.74 ± 0.92, 0.65 ± 0.95 and 0.68 ± 0.94 per cell, respectively.

### Ultrasound xenograft imaging

**Fig 6:**
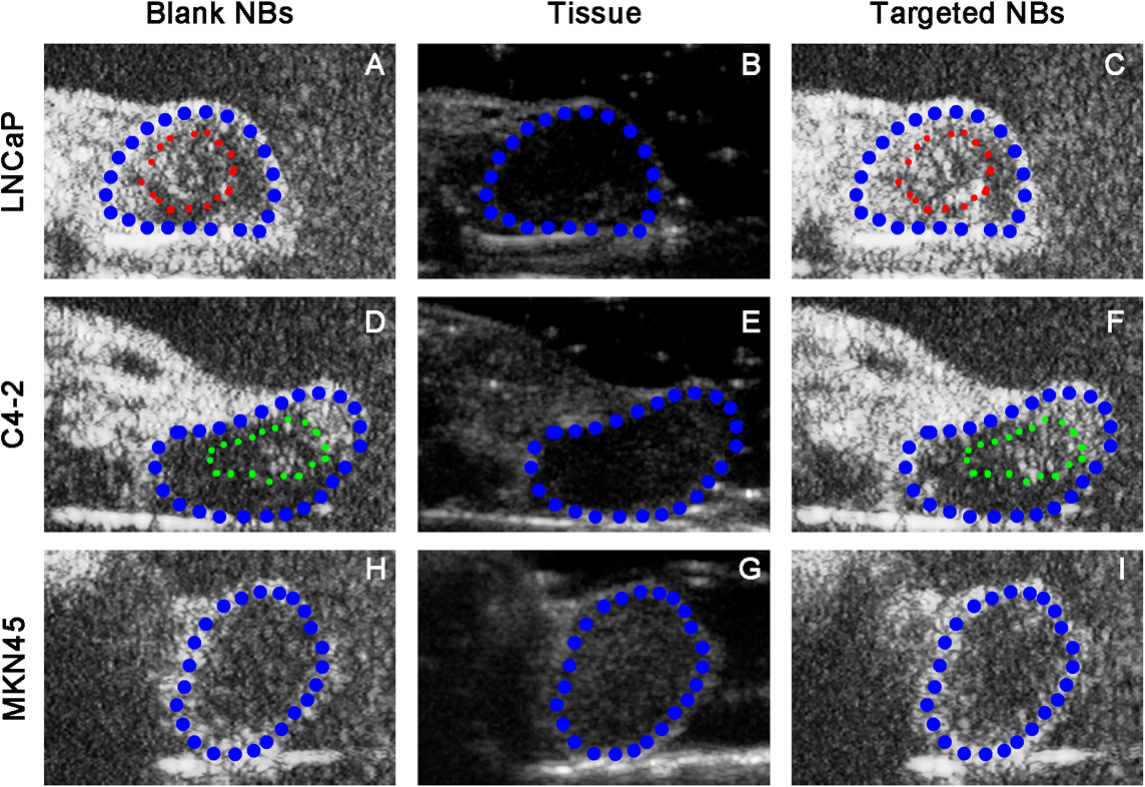
Imaging results of the Targeted NBs in tumor-bearing nude mice at the peak nanobubble level. Panels B, E and G show the classical cross-sections of 3 types of xenografts under B-mode ultrasonography. Panels A, D and H show the binding of Blank NBs to the xenografts at the peak intensity. Panels C, F and I show the binding of Targeted NBs to the xenografts at the peak intensity. The ultrasonographic images in both the LNCaP and C4-2 xenografts revealed that the imaging intensity was apparently higher than the imaging intensity achieved with the Blank NBs at the peak nanobubble level. In the MKN45 xenografts, the imaging results of the Targeted NBs and Blank NBs were comparable at the peak nanobubble level. Of all, blue areas represent the exnografts, while red and yellow areas respectively represent the different imaging areas in LNCaP and C4-2 exnografts.

Qlab8.1 software was used to analyze and compare the imaging indicators (arrival time, peak time, peak intensity and enhanced duration) of the Targeted NBs and Blank NBs in the 3 xenograft groups (Fig 6 and Table 1). In the prostate cancer xenografts (LNCaP and C4-2), the results revealed that the peak intensity values (P values, 0.003 and 0.002, respectively) and enhanced durations (P values, 0.001 and 0.004, respectively) of the Targeted NBs were significantly higher and longer, respectively, than in the Blank NBs. However, the arrival times and peak times were indistinguishable between the 2 types of nanobubbles. In the control MKN45 gastric cancer xenograft group, the Targeted NBs and Blank NBs exhibited no clear differences with respect to the 4 indicators. A comparison of the 3 types of xenografts with respect to the targeted nanobubble imaging properties revealed that the C4-2 xenografts exhibited significantly different peak intensity values, enhanced durations, arrival times and peak times compared the MKN45 xenografts, with respective P values of 0.024, 0.007, 0.000 and 0.050. Moreover, the LNCaP xenografts also displayed significantly different arrival times and peak values when compared with those of MKN45 xenografts (P values of 0.000 for both), although the remaining 2 indicators were not significantly different. Therefore, using ultrasonography imaging with Targeted NBs, both the androgen-dependent LNCaP prostate cancer cells and androgen-independent C4-2 cells manifested greater peak values and later arrival times than the control gastric cancer xenografts. Furthermore, when the LNCaP and C4-2 xenografts were compared, the arrival time (P = 0.026), peak time (P = 0.005) and peak value (P = 0.008) differed significantly whereas the enhanced duration was not significantly different (P = 0.261). However, the small differences (only less than one second) in the arrival time and time to peak between two tumors or two kinds of contrast agents are not easy to be observed, so the peak value and imaging duration should be the focus of most concern.

**Table 1:** Comparative ultrasonography indicator analysis of the Targeted NBs and Blank NBs in 3 types of xenografts.

Contrast Agents		Arrival Time (s)	Time to Peak (s)	Peak Intensity (dB)	Imaging Duration (min)
LNCaP tumor	Blank NBs	2.40±0.03	9.41±0.11	19.62±0.44	20.20±0.28
	Targeted NBs	2.42±0.09^#^ ^,^ ^+^	9.40±0.22^+^	21.47±0.60^*^ ^,^ ^#^ ^,^ ^+^	22.58±0.74^*^
C4-2 tumor	Blank NBs	2.30±0.02	9.06±0.10	19.20±0.40	21.29±0.52
	Targeted NBs	2.32±0.07^#^	9.05±0.12^#^	20.52±0.44^*^ ^,^ ^#^	23.00±0.33^*^
MKN45 tumor	Blank NBs	2.20±0.02	9.40±0.12	18.46±0.29	22.14±0.56
	Targeted NBs	2.21±0.03	9.38±0.10	18.50±0.33	22.23±0.53

The data are presented as the means ± SD (n = 5).

^*^P <0.05 indicates the level of significance compared with Blank NBs in the same types of xenografts;

^#^P <0.05 indicates the level of significance compared with targeted NBs in the gastric cancer xenografts (MKN45);

^+^P <0.05 indicates the level of significance compared with targeted NBs in the C4-2 xenografts.

## Discussion

Currently, prostate cancer is common and severely compromises the health of elderly men, often leading to death [19,20]. Although transrectal ultrasonography has become a routine examination tool that plays an important role in disease biopsy, monitoring and treatment, clinical studies have revealed that this procedure is limited by insufficient sensitivity and specificity [21–23]. The emerging field of molecular ultrasound imaging integrates ultrasonography, molecular biology and other disciplines and thus introduces a new avenue by which to diagnose and treat tumors; it also provides the possibility of specific prostate cancer imaging at a molecular level and corresponding early diagnoses [24]. Currently, the construction and design of prostate-cancer-targeted microbubbles have mostly focused on target molecules implicated in angiogenesis, including targeted microbubbles that carry vascular endothelial growth factor receptor type 2 (VEGFR2) antibody. However, this design has 2 apparent drawbacks: i) the mostly micron-scale contrast agents have poor permeability and cannot travel across tumor blood vessels to enter the interstitial spaces and therefore targeting bubbles cannot directly adhere to prostate cancer cells; ii) this method has a low specificity that leads to an inability to conduct specific prostate cancer tissue imaging [25]. The EPR effort of tumors makes it theoretically possible to use nanobubbles in molecular imaging of the tumor extravascular matrix and tumor parenchymal cells. Previously, using light and electron microscopy, we demonstrated that nanobubbles with an average diameter of 435.20 ± 60.53 nm could pass through the vascular endothelial gaps in xenografts, thus providing an experimental basis on which to achieve targeted imaging of tumor extravascular structures [26]. Meanwhile, this method also takes full advantage of some of the attributes of nanobubbles, such as the relatively large surface area, strong adhesion capacity and long lasting imaging *in vivo*.

PSMA is a prostate cancer biomarker with a higher specificity and sensitivity than other similar molecules. PSMA is particularly highly expressed in hormone-refractory prostate cancers and prostate cancer metastases [27]. Additionally, the extracellular region of PSMA, which comprises 707 amino acids, accommodates multiple antigenic epitopes. Hence, PSMA has become a research focus with respect to immune-targeted tumor therapies and molecular tumor imaging [28,29]. Sanna *et al*. conjugated the urea-based PSMA inhibitor DCL to the microbubble envelope component poly(lactic-co-glycolic acid-polyethylene glycol (PLGA-PEG), thus generating a targeted ultrasound contrast agent to be used for prostate cancer cell binding evaluations *in vitro* [30]. In our previous study, the adopted monoclonal antibody was immunogenic, which, in conjunction with the large molecular weights of the antibody-paticle complexes, led to poor tissue penetration. Consequently, after venous administration, the concentration in the targeted area was low. This problem severely limited the greater clinical application of microbubbles for targeted diagnostic and imaging modalities. Alternatively, specific small-molecule peptides are often used in molecular imaging studies. Although these compounds are easily synthesized and feature low molecular weights, high levels of tissue infiltration and low immunogenicity, they also exhibit problems such as short half-lives (proneness to hydrolysis and renal clearance) and volatile affinities, which significantly damage the stability of short peptide-bearing targeted nanobubbles. In contrast, nanobodies are highly stable and exhibit high antigen affinities. Moreover, they have very low immunogenicity, as evidenced by animal experimental results in which no humoral or cellular immune responses were detected after repeated administration [31]. Therefore, the various properties of nanobodies have provided convincing evidence regarding the feasibility of targeting and concentrating nanobubbles within target tissues *in vivo*. Although nanobodies have reportedly been applied in molecular imaging studies, including some molecular nuclear medicine applications [32–34], the application of this technology into the field of ultrasonography has been limited to ultrasound microbubbles[35]; to our knowledge, few researches regarding nanobody-labeled nanobubbles have been reported in this field.

Hence, we used a biotin-avidin system to integrate the advantages of nanobubbles and nanobody by generating nanobubbles that harbored PSMA nanobody. The average particle diameter was 487.60 ± 33.55 nm, which was significantly smaller (P = 0.003) than our previously produced nanobubbles that carried PSMA monoclonal antibodies (644.30 ± 55.85 nm). The results indicated that the newly developed targeted nanobubbles were superior to the previously generated particles with respect to minimization and safety [8,35]. In *in vitro* targeting studies, PSMA nanobody-carrying nanobubbles could specifically adhere to prostate cancer cells (including androgen-dependent LNCaP cells and androgen-independent C4-2 cells). In the targeted *in vivo* prostate cancer xenograft imaging experiment, the Targeted NBs displayed significantly higher peak intensity values and significantly longer enhanced durations than did Blank NBs, which was very important for monitoring treatment response. Compared with the control MKN45 gastric cancer xenografts, prostate cancer xenografts displayed significantly later arrival times and significantly higher peak intensity values, suggesting that our prepared Targeted NBs binded specifically *in vivo* compared to the Blank NBs. Moreover, these particles exhibited a distinctive contrast-enhancing effect and their imaging properties in prostate cancer xenografts included a high peak value and a later arrival time. However, we should concentrate on the differences in peak intensity and imaging duration in view of easy observation.

Notably, PSMA nanobody from an immune antibody library panning was reported to display a high affinity (K_D_ = 1 × 10^−8^ mol/L^-1^) [36]. Although our nanobody selected during the panning of a non-immune nanobody library, has a slightly lower affinity (K_D_ = 5.19× 10^−7^ mol/L^-1^), our previous experiments demonstrated that nanobody phage clones specifically binding to PSMA-positive LNCaP cells could be identified and that the selected nanobody could bind PSMA in ELISA [17]. Moreover, selection of specific nanobodies from a natural phage-based nanobody library could avoid active immunization of camelids or certain cartilaginous fishes [37]. Furthermore, although our study revealed that minimized targeted nanobubbles could bind to prostate cancer cell targets, thus boosting the xenograft signal levels *in vivo*, future studies will be needed to elucidate the events and mechanisms by which these nanobubbles integrate with the prostate cancer targets and to examine whether these targeted nanobubbles might influence cancer signaling pathways via binding to PSMA.

## Conclusions

In summary, we conducted a constructive attempt to apply genetically engineered antibodies to molecular ultrasonography in which we constructed and prepared PSMA nanobody-coupled ultrasound nanobubbles. Subsequently, we examined the targeted binding affinities of these nanobubbles for prostate cancer cells and investigated the effects of specific imaging in a nude mouse prostate cancer xenograft model. Our results have not only provided experimental methods with which to study nanobubbles in the context of molecular tumor ultrasonography but have also provided a basis from which to experiment with drug-carrying targeted nanobubbles for prostate cancer treatment.
